# Exploring the key genes and signaling transduction pathways related to the survival time of glioblastoma multiforme patients by a novel survival analysis model

**DOI:** 10.1186/s12864-016-3256-3

**Published:** 2017-01-25

**Authors:** Yuan Xia, Chuanwei Yang, Nan Hu, Zhenzhou Yang, Xiaoyu He, Tingting Li, Le Zhang

**Affiliations:** 1 College of Computer and Information Science, Southwest University, Chongqing, 400715 People’s Republic of China; 2 Systems Biology, the University of Texas MD Anderson Cancer Center, Houston, USA; 3 Breast Medical Oncology, the University of Texas MD Anderson Cancer Center, Houston, USA; 4 Cancer Center, Research Institute of Surgery, Daping Hospital, Third Military Medical University, Chongqing, 400042 People’s Republic of China; 5 Chongqing Zhongdi Medical Information Technology Co., Ltd, Chongqing, 401320 People’s Republic of China; 6 College of Mathematics and Statistics, Southwest University, Chongqing, 400715 People’s Republic of China

**Keywords:** Least absolute shrinkage and selection operator (Lasso), Sure independence screening (SIS), Cox proportional hazards model (Cox), Glioblastoma multiforme (GBM), Signaling transduction pathway

## Abstract

**Background:**

This study is to explore the key genes and signaling transduction pathways related to the survival time of glioblastoma multiforme (GBM) patients.

**Results:**

Our results not only showed that mutually explored GBM survival time related genes and signaling transduction pathways are closely related to the GBM, but also demonstrated that our innovated constrained optimization algorithm (CoxSisLasso strategy) are better than the classical methods (CoxLasso and CoxSis strategy).

**Conclusion:**

We analyzed why the CoxSisLasso strategy can outperform the existing classical methods and discuss how to extend this research in the distant future.

## Background

Glioblastoma multiforme (GBM) is the most common and malignant brain tumor [1–3]. Since GBM is high invasive and is mixed together with the healthy brain tissue, it is almost impossible to remove the tumor without causing serious consequences [4]. Moreover, GBM is very easy to relapse [5, 6]. The median survival and progression free survival time of GBM are 14.6 and 6.9 months, respectively. And the 5 year survival rate was 9.8 %[7]. Previous studies [8–10] indicated that gene mutation is one of the most important factors for GBM development. Therefore, gene expression analysis can not only be used to discover the underlying abnormality of gene expression associated with the GBM gene mutation, but also be employed to discover gene signatures which could help us to investigate the related signaling transduction pathways. Results from the pathway analysis can lay the foundation for the GBM cancer targeted drug research in the future.

As one of the important survival analysis methods, the cox proportional hazards model [11] is broadly employed to investigate the connections between various covariates and the length of life. However, the classical cox proportional hazards model [12] can only process such survival data that the dimension of the factors (P) are less than the number of samples (N) [13] (we call it as P < <N type of data), but it is not able to handle the survival data that the dimension of the factors are greater than the number of samples such as the gene expression data [13] (we call it as P> > N type of data). To process P> > N type of data, Tibshirani et al., [14] integrated the Lasso algorithm, one of the constrained optimization methods, into the classical Cox proportional hazards model [15] to select the key predictors. However, Fan et al., [16] pointed out if the number of predictors is much greater than the sample size (P> > N), a pre-cleaning step by a computationally expedient screening procedure is often preferred to increase the accuracy of the algorithm. Thus, Fan et al., [16] developed the sure independence screening (SIS) method by fitting marginal Cox regression models for each covariate and screening out several covariates by a pre-specified threshold. Nevertheless, reported by Hong et al., [17], marginal screening may encounter the difficulty in identifying these hidden and jointly important variables to incur false negatives. Therefore, Hong et al., [17] proposed a conditional SIS method to explore the potential predictors for the regular linear system, but not consider the survival data. On the other hand, developing a systematic approach to identify the target generic drug for the cancer treatment already becomes a popular research field [18, 19]. However, to our knowledge, there is no recent research discussing the incoherent connection between survival time and the target generic drug in detail.

**Fig. 1:**
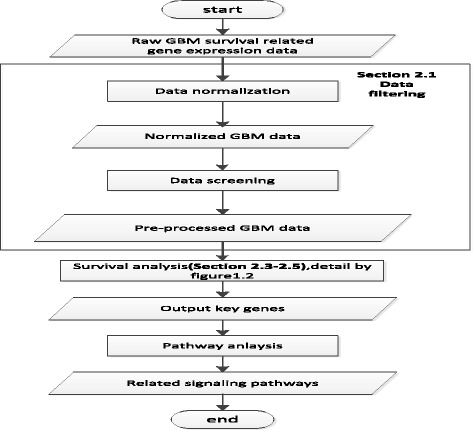
Flow chart of the gene and signaling transduction pathway platform

To overcome the shortcomings of these previous research, this study proposed a multi-scale genes and signaling transduction pathways exploration platform (Fig. 1) with the following three innovations. Firstly, we innovatively analyzed the clinical GBM gene expression and survival time data [20] to investigate the incoherent relation between the signatures of genes and the survival time of GBM patient. Secondly, we not only integrated the constrained optimization method such as Lasso [15] into classical Cox proportional hazards model [13] to explore survival time related key genes by processing the P> > N type of data, but also used the SIS algorithm to improve the predictive accuracy. Thirdly, we employed KOBAS database [21] and hypergeometric test [22] to investigate the correlated GBM signaling transduction pathways regarding the explored survival time related key genes. And then, these survival time related signaling transduction pathway could help us to bridge the relation between the targeted drugs and the survival time for GBM patients.

The clinical GBM gene expression and survival data set used in this study is downloaded from the Georgetown Database of Cancer G-DOC [20], which has 54,675 features (P) and 227 samples (N). To handle such a P> > N type of data, we developed the CoxSisLasso strategy. It firstly integrated constrained optimized methods such as Lasso into the classical cox regression model to select the prior genes with potentially great impact on the patients’ survival time. Secondly, conditioned on these genes selected by Lasso, conditional SIS method [23] is used to re-select the possible genes from these genes screened out in the first step. To bridge the relation between the targeted drugs and the survival time for GBM patients, we employed the KOBAS [21] application and the explored GBM survival time related key genes to investigate which signaling transduction pathways closely correlate with the GBM survival time.

In general, this study developed a multi-scale genes and signaling transduction pathways exploration algorithm that can not only investigate the molecular mechanism between the key genes and cancer patients’ survival time, but also employ hypergeometric distribution based database (KOBAS) to look for the related signaling pathways in the proteomics level for the future targeted cancer therapy [24, 25]. Manually-reviewed experimental evidences showed that mutually explored GBM survival time related genes [26–38] and signaling transduction pathways [39–52] are closely related to the GBM. In addition, the research results demonstrate that our proposed CoxSisLasso strategy has the best predictive power and model fitting capacity compared to the CoxLasso and CoxSis strategy developed by Tibshirani et al.,[14] and Fan et al., [16], respectively. Finally, we theoretically analyze why the CoxSisLasso strategy outperforms CoxLass and CoxSis and discuss the further research.

## Methods

### Materials

We used a multi-study microarray database of GBM expression profiles (*n* = 227) from the Georgetown Database of Cancer G-DOC [20], based on the Affymetrix U133 plus 2.0 GeneChip microarray platform. The microarray datasets of GBM are listed by Table 1.

**Table 1:** The illustration of microarray datasets of GBM

Data set name	Source site	Affymetrix platform	Sample size
GBM	DANA-FARBER CANCER INSTITUTE	HG-U133_Plus_2	2
GBM	NABTT/H. LEE MOFFITT CANCER CENTER	HG-U133_Plus_2	30
GBM	HENRY FORD HOSPITAL (RETRO)	HG-U133_Plus_2	62
GBM	M. D. ANDERSON CANCER CENTER	HG-U133_Plus_2	6
GBM	MSKCC/NEW YORK	HG-U133_Plus_2	2
GBM	NABTT/HENRY FORD HOSPITAL	HG-U133_Plus_2	7
GBM	NABTT/JOHNS HOPKINS	HG-U133_Plus_2	2
GBM	NIH NEURO-ONCOLOGY BRANCH	HG-U133_Plus_2	48
GBM	TJU	HG-U133_Plus_2	30
GBM	UCLA SCHOOL OF MEDICINE	HG-U133_Plus_2	7
GBM	UCSF	HG-U133_Plus_2	17
GBM	UNIV OF PITTSBURGH	HG-U133_Plus_2	9
GBM	UNIVERSITY OF WISCONSIN	HG-U133_Plus_2	5

### Data filtering

The original microarray datasets are normalized and preprocessed by R software package [53]. After preprocessing step, there are 227 samples and 54,675 genes left in the data matrix. Next, the interquartile range (IQR) threshold [54] is employed to screen out the genes with small variance value. After that, there are only 227 samples and 10,992 genes left in the GBM gene expression and survival time data matrix.

### Cox proportional hazards model

Survival analysis [11, 55] works for the analysis of time duration until one or more events happen. As one of the widespread used survival analysis methods, the Cox proportional hazards model [13] is used to analyze the time-to-event data with both censored data and covariates, which assumes a semi-parametric form for the hazard as Eq. 1.

1 $$ {h}_i(t)={h}_0(t) \exp \kern0.5em \left({x}_i^T\beta \right) $$

where *h*_*i*_(*t*) is the hazard for patient *i* at time t, *h*_0_(*t*) is a shared baseline hazard function, *β* is an unknown p-dimensional regression coefficient vector and *x*_*i*_ is a vector of potential predictors for the *i* ^*th*^ individual. Based on the available samples, the estimator of the unknown parameter coefficients $\widehat{\beta}$, can be obtained by maximizing the log-partial likelihood function as Eq. 2

2 $$ \widehat{\beta}= \arg \max \log PL= \arg \max {\displaystyle \sum_{k\in D}\left[{x}_k^T\beta - \log \left({\displaystyle {\sum}_{j\in {R}_k} \exp}\left({x}_j^T\beta \right)\right)\right]} $$

where *D* is the set of indices of the events and *R*_*k*_ denotes the set of indices of the individuals at risk at time *t*_*k*_.

Since this study encounters the P> > N type of data, it is impossible to employ classical Cox proportional hazard regression method [13] to analyze the GBM gene expression data matrix directly. Therefore, the following sections propose three variable selection strategies to obtain the sparse regression coefficient.

### Combined Cox and Lasso (CoxLasso) strategy

To obtain the sparse solution for the parameter *β* in the Cox proportional hazards model (Eq. 1), we have to integrate constrained optimization methods such as Lasso proposed by Tibshirani et al.,[14] into classical Cox proportional hazards model to minimize the negative log partial likelihood subject to the sum of the absolute values of the parameters being bounded by a constant as Eq. 3.

3 $$ \widehat{\beta}= \arg \min -\left\{{\displaystyle \sum_{k\in D}\left[{x}_k^T\beta - \log \left({\displaystyle {\sum}_{j\in {R}_k} \exp}\left({x}_j^T\beta \right)\right)\right]}\right\}\kern0.75em \mathrm{subject}\ \mathrm{t}\mathrm{o}\kern0.5em {\displaystyle \sum_{j=1}^p\left|{\displaystyle {\beta}_j}\right|}\le t $$

**Fig. 2:**
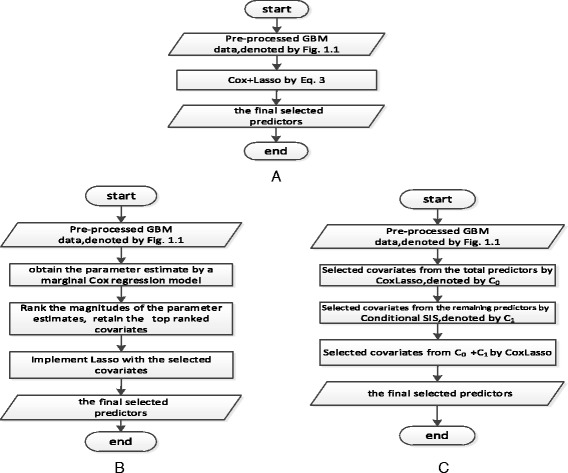
Flow chart of the strategy **a** CoxLasso, **b** CoxSis and **c** CoxSisLasso

It is equivalent to the following optimization problem

4 $$ \widehat{\beta}= \arg \min -\left\{{\displaystyle \sum_{k\in D}\left[{x}_k^T\beta - \log \left({\displaystyle {\sum}_{j\in {R}_k} \exp}\left({x}_j^T\beta \right)\right)\right]}\right\}+\lambda {\displaystyle \sum_{j=1}^p\left|{\beta}_j\right|} $$

where *λ* is the tuning parameter to control the sparsity of the estimator. This research used the R package tool glmnet developed by Friedman et al.,[56] to implement the combined Cox and Lasso (CoxLasso) strategy (Fig. 2a) by using cross validation to choose the tuning parameter.

### Combined Cox and SIS (CoxSis) strategy

Though directly integrating Lasso method into Cox model can process P> > N type of data, it may encounter problems with speed, stability, and accuracy, once the dimension of the covariates is ultra-high [23] . Therefore, it is often preferred to employ a simple and computationally efficient screening procedure to reduce the data dimensionality to a moderate size before using Lasso method. The combined Cox and SIS (CoxSis) strategy is illustrated by the following steps:

- Step 1: Fit a marginal Cox regression model for each covariate *x*_*m*_ to obtain ${\widehat{\beta}}_m$ by Eq. 5.1
  5.1 $$ {\widehat{\beta}}_m= \arg \max {\displaystyle \sum_{k\in D}\left[{x}_{km}{\beta}_m- \log \left({\displaystyle {\sum}_{j\in {R}_k} \exp}\left({x}_{jm}{\beta}_m\right)\right)\right]} $$
- Step 2: Rank the magnitudes of ${\widehat{\beta}}_j,j=1,2,\dots, p$ in decreasing order and keep the number of *d* top ranked covariates.
- Step 3: Denote the index of selected covariates by Θ. Implement Lasso with the selected *d* covariates by minimizing Eq. 5.2
  5.2 $$ \underset{\beta_{\Theta}}{ \min}\left\{-{\displaystyle \sum_{k\in D}\left[{x}_{k,\Theta}^T{\beta}_{\Theta}- \log \left({\displaystyle {\sum}_{j\in {R}_k} \exp}\left({x}_{j,\Theta}^T{\beta}_{\Theta}\right)\right)\right]}+\lambda {\displaystyle \sum_{j\in {\beta}_{\Theta}}\left|{\beta}_j\right|}\right\} $$

This study employs R package of SIS developed by Fan et al., [16] to implement the combined Cox and SIS (CoxSis) strategy (Fig. 2b).

### Combined Cox, SIS and Lasso (CoxSisLasso) strategy

Recently, Barut et al., [57] proposed a conditional screening approach (Conditional SIS) to enhance the accuracy of SIS by using the prior knowledge of the key factors to select the predictors. Regarding to our P> > N type of data and the limitation of Lasso method in the stability and accuracy, this study proposed a combined Cox, SIS and Lasso (CoxSisLasso) strategy (Fig. 2c) to increase the predictive accuracy of the model as follows:

- Step 1: Implement Lasso for the data. Denote the index of selected covariates with Lasso by *C*_0_.
- Step 2: Conditioned on the selected subset of covariates *C*_0_, for each covariate *x*_*m*_, *m* ∉ *C*_0_, fit the following Cox regression model by maximizing Eq. 6
  6 $$ {\widehat{\beta}}_m=\underset{\beta_m}{ \arg \max}\left\{{\displaystyle \sum_{k\in D}\left[{x}_{k,{C}_0}^T{\beta}_{C_0}+{x}_{k,m}{\beta}_m- \log \left({\displaystyle {\sum}_{j\in {R}_k} \exp}\left({x}_{j,{C}_0}^T{\beta}_{C_0}+{x}_{j,m}{\beta}_m\right)\right)\right]}\right\} $$
- Step 3: For a given threshold *γ*, keep the variables *x*_*m*_, *m* ∉ *C*_0_ if $\left|{\widehat{\beta}}_m\right|\ge \gamma$. Denote ${C}_1=\left\{m\notin {C}_0,\left|{\widehat{\beta}}_m\right|\ge \gamma \right\}$. Then the augmented selected predictors are *C*_0_ ∪ *C*_1_.
- Step 4: Implementing Lasso with the covariates in the set *C*_0_ ∪ *C*_1_ to select the final predictors.

For the threshold *γ*, Barut et al.,[57] proposed two procedures by controlling FDR and random decoupling to choose the proper level of threshold. Motivated by Zhao and Li [23], this study sets the threshold *γ* = 1/*p*, and p is the total number of all the covariates. Once the p-value of the Z-test of the covariate *x*_*m*_, *m* ∉ *C*_0_ is less than the *γ*, we keep it as one of the important predictors.

### Investigate potential signaling pathway regarding to the candidate genes related to the GBM survival time

After obtaining the explored GBM survival time related key genes by previous strategies, it is interesting for us to investigate which potential signaling pathways are closely related to these genes. And the potential pathways will be employed for the targeted drug therapy to treat the GBM cancer in the future.

KOBAS is a signaling transduction pathway database to identify statistically significantly enriched pathways using hypergeometric test [11]. In statistics, the hypergeometric test uses the hypergeometric distribution (Eq. 7) to calculate the statistical significance.

7 $$ p\left(X=k\right)=\frac{\left(\begin{array}{c}\hfill K\hfill \\ {}\hfill k\hfill \end{array}\right)\left(\begin{array}{c}\hfill N-K\hfill \\ {}\hfill n-k\hfill \end{array}\right)}{\left(\begin{array}{c}\hfill N\hfill \\ {}\hfill n\hfill \end{array}\right)} $$

where N is the population size, K is the number of success states in the population, n is the number of draws, k is the number of observed successes.

## Results

### The explored GBM survival time related key genes by CoxLasso, CoxSis and CoxSisLasso strategy, respectively

**Fig. 3:**
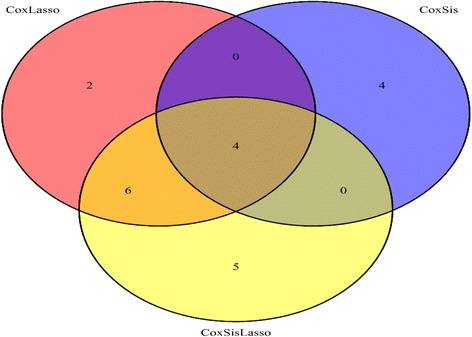
Venn plot for the explored GBM genes

Here, Table 2 shows the explored GBM survival time related key genes by CoxLasso, CoxSis and CoxSisLasso strategy, respectively. Also, the Venn plot (Fig. 3) indicates there are four common genes (AEBP1, GDNF, IL17RC and EIF3A) mutually selected by these three strategies, which closely correlate with the survival time of GBM patient validated by the manually-reviewed experimental evidences [26–38].

**Table 2:** The explored genes for strategy CoxLasso, CoxSis and CoxSisLasso

Method	Key genes
CoxLasso	ARIH2, ZNF786, AEBP1, FOXG1, INTS1, GDNF, CUTC, SGCD, CCM2, IL17RC, EIF3A, CBLN1
CoxSis	YAP1, TRAF3IP2, AEBP1, GDNF, EAF2, ST5, IL17RC, EIF3A
CoxSisLasso	ARIH2, ZNF786, AEBP1, FOXG1, INTS1, GDNF, SGCD, IL17RC, EIF3A, CBLN1, SLC35D1, ELOVL2, CDCA7L, SNTB1, TELO2
CoxLasso, CoxSis and CoxSisLasso	AEBP1, GDNF, IL17RC, EIF3A

Firstly, AEBP1 (Adipocyte enhancer binding protein 1) was discovered as a transcriptional repressor [26]. It not only expresses at different levels in different organ and tissue types and its expression is relatively strong in brain [27], but also it can interact with tumor suppressor protein PTEN and inhibit its tumor-suppressing function [28]. AEBP1 can also negatively regulate IkB, resulting in the up-regulation of NF-kB and enhanced inflammatory response [29]. It is well known that both PTEN and NF-kB, closely related to the AEBP 1, are important players in GBM cancer progression. Moreover, previous research identified several genomic targets of AEBP1 playing vital roles in the survival of glioma cells [30].

Secondly, GDNF is a Glial Cell derived neurotrophic factor which promotes survival of neurons [31]. GDNF is not only identified as an important factor in macrophage infiltration into GBM, contributing to GBM progression [32], but also it can promote glioma cell invasion through its receptors that are present on invasive GBM cells [33].

Thirdly, EIF3A (Eukaryotic translation initiation factor subunit 3A) is not only expressed in all tissue types in human body and its expression is up-regulated in some type of cancers [34], but also it is important in regulating the expression of proteins involved in DNA repair pathway which is essential in drug sensitivity and resistance in cancer treatment [35, 36]. Especially, EIF3A is found to be overexpressed in some glioma patients [37].

Fourthly, Inlerleukin-17 receptor C (IL17RC) is a key molecule mediating interleukin 17 signaling. It is important in immune response and inflammation which are important in GBM progression [38].

### Predictive performance comparison of survival time for each strategy

This study employs the idea of time-dependent receiver operating characteristic curve (ROC) for the censored data and the area under the curve (AUC) [58, 59] to quantify the predicative accuracy for each strategy, when the outcome of interest is the survival time. The ROC curve depicts the sensitivity (Eq. 8.1) versus 1-specificity (Eq. 8.2) at each time t for any risk score function *x* ^*T*^ *β*

8.1 $$ sensitivity\left(c,t\Big|{x}^T\beta \right)= \Pr \left\{{x}^T\beta >c\Big|\delta (t)=1\right\} $$

8.2 $$ specificity\left(c,t\Big|{x}^T\beta \right)= \Pr \left\{{x}^T\beta \le c\Big|\delta (t)=0\right\} $$

with *c* being the cut-off value and *δ*(*t*) is the event indicator at time t.

**Fig. 4:**
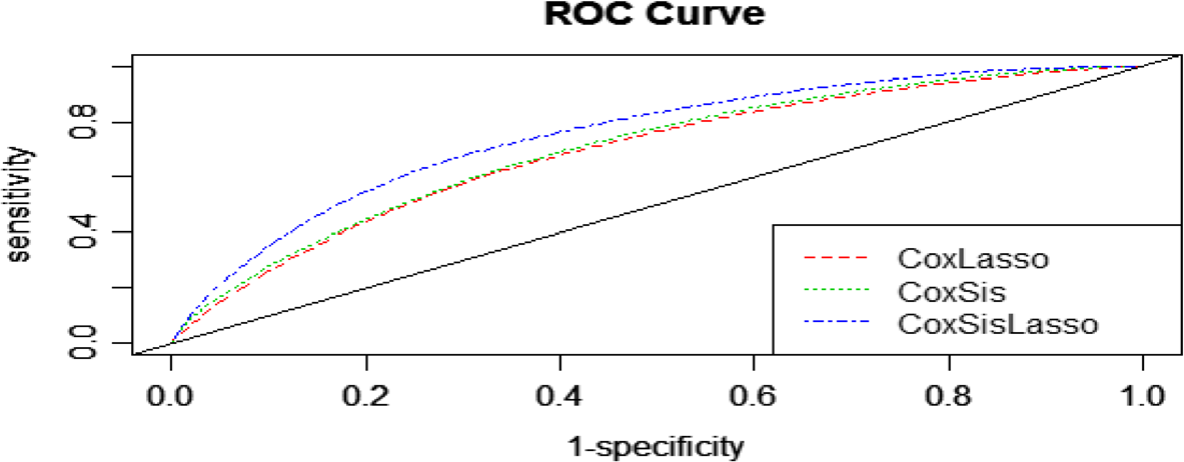
ROC curves for strategy CoxLasso, CoxSis and CoxSisLasso

**Fig. 5:**
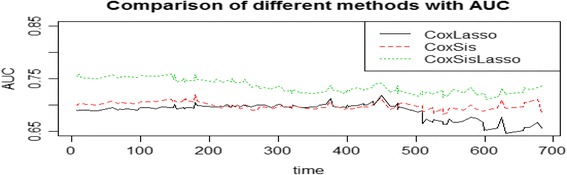
AUCs for strategy CoxLasso, CoxSis and CoxSisLasso

**Fig. 6:**
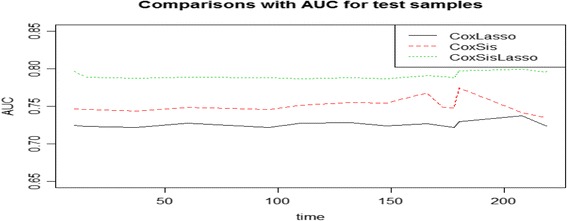
AUCs for test samples for strategy CoxLasso, CoxSis and CoxSisLasso

Figures 4 and 5 depicts the ROC curve at a specific predicted time 30 and AUC over a period of time respectively to quantify the performance of the three strategies to predict the survival time of the GBM patients. It demonstrates that CoxLasso and CoxSis strategy shares the similar predictive performance, whereas our proposed CoxSisLasso strategy has the best predictive accuracy since it not only has the greatest value of the sensitivity and 1-specificity (Fig. 4), but also the largest AUC value (Fig. 5). Furthermore, to assess the generalization ability of the proposed model, we randomly select 120 samples as the training samples and the rest 68 samples as the test samples. Figure 6 shows the AUCs of the three strategies for the testing samples. Both Figs. 5 and 6 turn out that our proposed CoxSisLasso method provides the largest AUC value with the best performance.

### Model fitting performance comparison for each strategy

Table 3 summarizes the Cox regression results with the key genes selected by three strategies. R^2^ is the statistic of the goodness of fit measure [60]. The concordance index [61] is a valuable measure of model discrimination in analyses involving survival time data. Greater R^2^ and concordance index value imply better model fitting performance. Table 3 shows that R^2^ and concordance index value of CoxSisLasso strategy (Table 3C) outperforms the other two (Table 3A & B). Moreover, by comparing results of Table 3C (CoxSisLasso) with the results of Table 3A (CoxLasso) and Table 3B (CoxSis), we found that CoxSisLasso not only can preserve the genes selected by the CoxLasso and CoxSis, but also it can introduce several statistically significant genes, which are potential for us to explore their relationships with GBM in the distant future.

**Table 3:** Model fitting results for strategy (A) CoxLasso, (B) CoxSis and (C) CoxSisLasso

Key genes	coef	exp(coef)	se(coef)	z	*p*-value
A					
ARIH2	0.28827	1.33412	0.20957	1.376	0.1690
ZNF786	0.73967	2.09524	0.31849	2.322	0.0202*
AEBP1	0.09910	1.10418	0.09315	1.064	0.2874
FOXG1	0.14722	1.15861	0.06712	2.193	0.0283*
INTS1	0.19661	1.21726	0.27385	0.718	0.4728
GDNF	−0.33054	0.71854	0.29059	−1.137	0.2553
CUTC	−0.03165	0.96885	0.27837	−0.114	0.9095
SGCD	0.12861	1.13724	0.20752	0.620	0.5354
CCM2	0.29707	1.34591	0.28104	1.057	0.2905
IL17RC	0.51024	1.66569	0.21579	2.364	0.0181*
EIF3A	−0.27131	0.76238	0.23337	−1.163	0.2450
CBLN1	−0.29685	0.74316	0.30079	−0.987	0.3237
R^2^ = 0.338, Concordance = 0.687					
B					
YAP1	−0.28804	0.74973	0.12372	−2.328	0.019902*
TRAF3IP2	−0.39514	0.67358	0.20318	−1.945	0.051805
AEBP1	0.33103	1.39239	0.09268	3.572	0.000354***
GDNF	−1.09305	0.33519	0.30549	−3.578	0.000346***
EAF2	−0.53363	0.58647	0.21472	−2.485	0.012949*
ST5	−0.26305	0.76870	0.26139	−1.006	0.314240
IL17RC	1.02690	2.79240	0.22954	4.474	7.69e-06***
EIF3A	−0.40494	0.66702	0.21963	−1.844	0.065216
R^2^ = 0.375, Concordance = 0.696					
C					
ARIH2	0.27310	1.31403	0.20654	1.322	0.186080
ZNF786	1.17873	3.25025	0.31923	3.692	0.000222***
AEBP1	0.20724	1.23027	0.09558	2.168	0.030151*
FOXG1	0.32694	1.38672	0.09262	3.530	0.000416***
INTS1	0.85607	2.35388	0.34015	2.517	0.011844*
GDNF	−0.48393	0.61636	0.35170	−1.376	0.168835
SGCD	−0.53359	0.58650	0.22601	−2.361	0.018233*
IL17RC	1.23644	3.44332	0.23552	5.250	1.52e-07***
EIF3A	−0.08224	0.92105	0.22885	−0.359	0.719339
CBLN1	−1.06495	0.34474	0.38400	−2.773	0.005548**
SLC35D1	−0.44547	0.64052	0.21196	−2.102	0.035579*
ELOVL2	−0.16161	0.85077	0.08395	−1.925	0.054210
CDCA7L	−0.38939	0.67747	0.11097	−3.509	0.000450***
SNTB1	−0.61372	0.54133	0.16747	−3.665	0.000248***
TELO2	−1.28721	0.27604	0.48148	−2.673	0.007507**
R^2^ = 0.515, Concordance = 0.747					

Significance codes: 0 ‘***’ 0.001 ‘**’ 0.01 ‘*’ 0.05

﻿

### The explored GBM survival time related signaling transduction pathways by CoxLasso, CoxSis and CoxSisLasso strategy, respectively

**Fig. 7:**
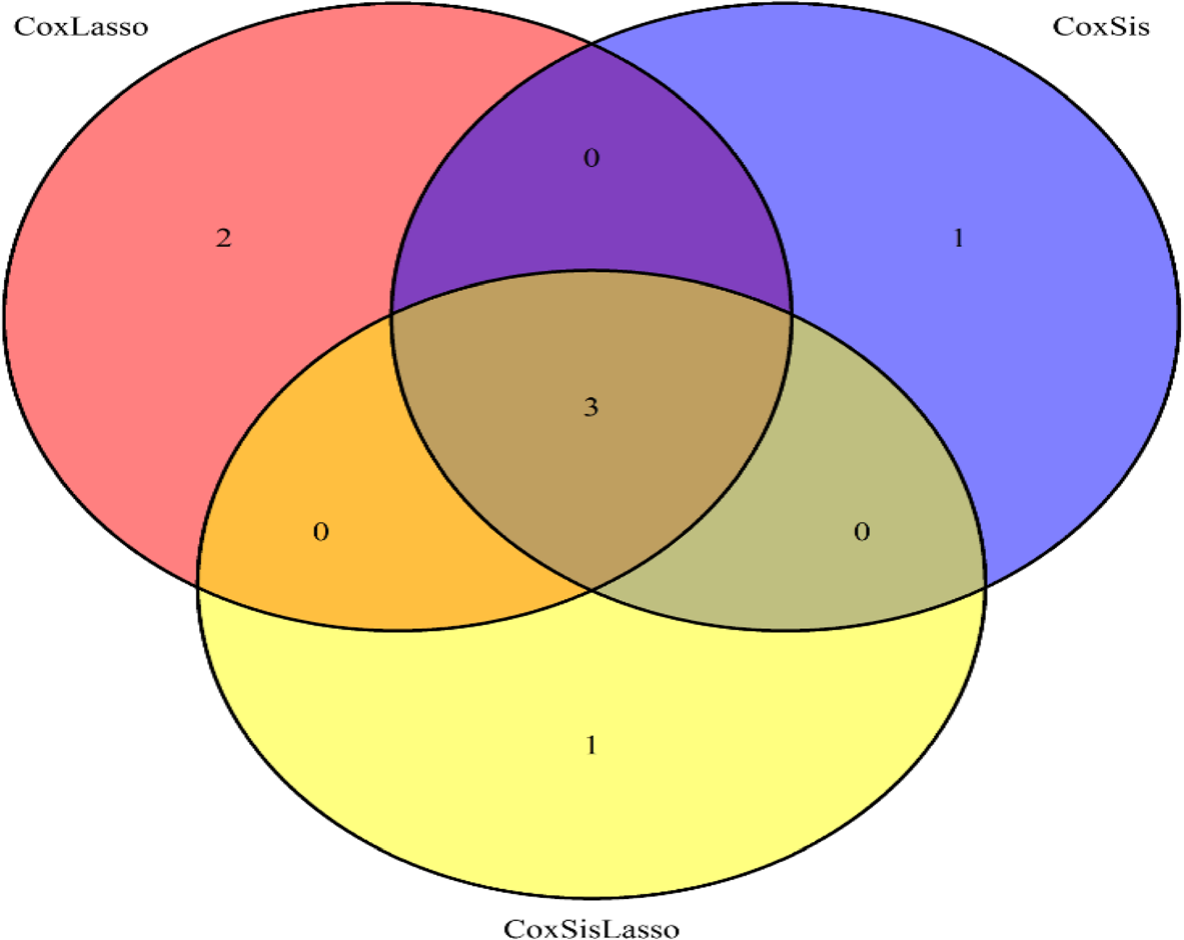
Venn plot for the GBM related signaling transduction pathways

Here, Table 4 lists the explored GBM related signaling transduction pathways by CoxLasso, CoxSis and CoxSisLasso strategy, respectively. Also, the Venn plot (Fig. 7) indicates three explored GBM related signaling transduction pathways mutually selected by these three strategies.

**Table 4:** The explored signaling transduction pathways for strategy CoxLasso, CoxSis and CoxSisLasso

Method	Key pathways
CoxLasso	1. internal ribosome entry pathway
	2. mtor signaling pathway
	3. TGF-beta signaling pathway
	4. p38 MAPK signaling pathway
	5. FoxO signaling pathway
CoxSis	1. Hippo signaling pathway
	2. internal ribosome entry pathway
	3. mtor signaling pathway
	4. TGF-beta signaling pathway
CoxSisLasso	1. internal ribosome entry pathway
	2. mtor signaling pathway
	3. TGF-beta signaling pathway
	4. Fanconi anemia pathway
CoxLasso, CoxSis and CoxSisLasso	1. internal ribosome entry pathway
	2. mtor signaling pathway
	3. TGF-beta signaling pathway

And then, we employed the manually-reviewed experimental evidences [39–52] to demonstrate that these mutually explored signaling transduction pathways closely correlate with the survival time of GBM patient as following.

Firstly, mTOR (Mammalian target of rapamycin) is an important mediator of phosphatidyl-inositol-3 kinase (PI3K) pathway. And previous research turned out that constitutive activation of PI3K signaling is found in the majority of GBM patients [39]. Moreover, PI3K-akt-mTOR axis plays essential role in cell growth and proliferation [40]. Signaling of mTOR pathway is vital for cancer cell growth and survival in GBM patients [41]. Currently mTOR pathway inhibitors are under active investigation in preclinical experiments and in clinical trials for GBM treatment [42].

Secondly, TGF-beta (Transforming growth factor beta) is a secreted cytokine which signals through specific receptors and exerts its effect via intracellular Smad family proteins [43]. TGF-beta pathway controls GBM cell proliferation [44]. Its signaling contributes to the maintenance of tumor-initiating cells in GBM [45]. TGF-beta pathway is also involved in tumor invasion and metastasis in GBM patients [46]. Inhibition of TGF-beta pathway signaling reduced GBM cell proliferation and invasion in preclinical cell-based assays [47]. TGF-beta pathway inhibitors showed promising results in improving GBM patient survival in clinical trials [48].

Thirdly, IRE (Internal Ribosomal Entry) pathway is involved in the synthesis of some proteins during which protein synthesis is initiated from a start codon near an IRE site rather than by scanning the Kozak sequence. This IRE pathway is used in the translation of many eukaryotic genes including growth factors such as VEGF, FGF2 and PDGF [49] and transcription factors such as c-myc and hypoxia induced factor [50, 51] . Indeed, upregulated expression of proto oncogene c-Jun in human GBM is mediated through a potent internal ribosomal entry site (IRES) in the 5′UTR of the c-Jun mRNA, and the upregulation of c-Jun contributes to the malignant properties of GBM cells [52].

## Discussion

This study developed a multi-scale gene and signaling transduction pathway exploration platform based on the classical Cox proportional hazard model [12], constrained optimization method [14–16] and hypergeometric test to analyze P> > N type of GBM gene expression and survival time data (Table 1). Compared to the previous research [14–16, 62], we proposed a novel CoxSisLasso strategy to investigate relationship between genes and GBM patients’ survival time in molecular level as well as used KOBAS database [21] to look for the survival time related signaling transduction pathways.

On the one hand, manually reviewed experimental evidences validate that both mutually explored key genes [26–38] (Table 2) and signaling transduction pathways [39–52] (Table 4) are closely related to GBM. On the other hand, since CoxLasso strategy may encounter problems with speed, stability, and accuracy for processing high dimensional data [23], the CoxSis strategy is developed by employing a simple and computationally efficient screening procedure to reduce the dimensionality of the data to a moderate size before using Lasso method based on the previous work of Fan et al., [16]. Though classic marginal screening approach based CoxSis is theoretically proved to be capable of selecting all important predictors [56], it is difficult to identify these hidden predictors which jointly correlate with the response variable but not marginally. For this reason, we proposed the CoxSisLasso strategy, which not only uses the CoxLasso strategy to obtain a prior set of important predictors, but also incorporates the SIS [16] approach to select the important predictors regarding to the previous results. Figure 5 and 6 turned out that CoxSisLasso strategy has the best predictive power and model fitting capacity than both CoxLasso and CoxSis.

## Conclusions

In general, this study innovatively developed a CoxSisLasso strategy to interrogate the connections between GBM gene expression and GBM patients’ survival time as well as employed the KOBAS database [21] and hypergeometric test [21] to investigate the incoherent signaling transduction pathways and the survival time of GBM patient. Though the research results demonstrated the advantages of our algorithm, the current research still has several shortcomings such as the theoretically proof for the CoxSisLasso strategy, simulation study for the gene and pathway selection platform and so on. In the distant future, we will not only need improve our current CoxSisLasso algorithm, but also will employ the related pathway analysis theory [63] to explore the GBM survival time related proteins for the target drug study.

## Abbreviations

AEBP1: Adipocyte enhancer binding protein 1
CoxLasso: Combined Cox and Lasso
CoxSis: Combined Cox and SIS
CoxSisLasso: Combined Cox, SIS and Lasso
EIF3A: Eukaryotic translation initiation factor subunit 3A
GBM: Glioblastoma multiforme
GDNF: Glial Cell derived neurotrophic factor
IL17RC: Inlerleukin-17 receptor C
IQR: Interquartile range
IRE: Internal Ribosomal Entry
IRES: Internal ribosomal entry site
mTOR: Mammalian target of rapamycin
PI3K: Phosphatidyl-inositol-3 kinase
SIS: Sure independence screening
TGF-beta: Transforming growth factor beta
